# Forecast horizon of multi-item dynamic lot size model with perishable inventory

**DOI:** 10.1371/journal.pone.0187725

**Published:** 2017-11-10

**Authors:** Fuying Jing, Zirui Lan

**Affiliations:** 1 School of Management and Economics, University of Electronic Science and Technology of China, Chengdu, China; 2 School of Economic and Management, Tianjin University of Science and Technology, Tianjin, China; University of Rijeka, CROATIA

## Abstract

This paper studies a multi-item dynamic lot size problem for perishable products where stock deterioration rates and inventory costs are age-dependent. We explore structural properties in an optimal solution under two cost structures and develop a dynamic programming algorithm to solve the problem in polynomial time when the number of products is fixed. We establish forecast horizon results that can help the operation manager to decide the precise forecast horizon in a rolling decision-making process. Finally, based on a detailed test bed of instance, we obtain useful managerial insights on the impact of deterioration rate and lifetime of products on the length of forecast horizon.

## 1. Introduction

In a multi-period, dynamic decision-making environment, forecast horizon is a period with the property that the data for periods beyond it are not required in order to determine the decisions of the first few periods. More formally, period *T* is referred to a forecast horizon if the data information (e.g. cost and demand) after period *T* does not affect the optimal decisions of the first *τ* periods in every *N*-period problem with *N* ≥ *T* + 1, period *τ* is the corresponding decision horizon (planning horizon). If *T* is a forecast horizon, decisions of the first *τ* periods for some optimum solution of the *T*-period problem are also optimum for each instance of the problem with larger than period *T*. The significance of forecast horizon *T* is immediate: it is unnecessary to forecast information after period *T*.

The earliest study of forecast horizons in operations management is due to Wagner and Whitin [1], who analyze a dynamic lot sizing (DLS) problem in the presence of setup costs, holding cost, deterministic time-varying demands. They demonstrate the optimality of Zero Inventory Property (ZIP) and use it to develop an efficient dynamic programming (DP) which solves the problem. They also investigate forecast horizon results. Afterwards forecast horizon has been widely applied in operations management [2–16]. Furthermore, the idea of forecast horizon has been applied to dynamic decision problems in the area of capacity expansion [17–18], machine replacement [19–20], plant location [21–22], cash balance [23–24], bond refunding [25]. Chand *et al*. [26] present an extensive classified bibliography of the forecast horizon literature. Since 2002, the research of forecast horizon incorporates the following aspects: operations management [27–34], game theory [35], reservoir operations [36–38].

In this paper we investigate forecast horizon in a multi-product DLS problem with age-dependent stock deterioration and inventory cost function and joint ordering. Our approach is motivated by Hsu [39], who points out that age-independent inventory cost structure in the conventional DLS models is reasonable for nonperishable products, but is not applicable to perishable products. He studies a new DLS model for perishable products where stock’s deterioration rates and inventory costs in each period depend on the age of the stock. Hsu [40] generalizes the new DLS model that backloggings are permitted. Chu *et al*. [41] extend the new DLS model under general economies of scale cost functions. Sargut and Isik [42] extend the study of Hsu [40] by considering production capacity. However, these studies on perishable products focus on single item. In addition, they do not investigate forecast horizons.

Our DLS problem belongs to a class of multi-product DLS problems with joint ordering. Due to the vast majority of research in this class, we discuss only a few articles that are related to our work. Kao [43] considers a multi-product DLS problem with joint and individual setup costs, and then present a DP algorithm for finding the optimal ordering policy. He also introduces a very simple heuristic procedure. Chung *et al*. [44] consider a coordinated replenishment DLS problem under all-units quantity discount and incremental quantity discount. They show their problem is NP-hard and present an effective polynomial time heuristic procedure for the incremental discount case. Li *et al*. [45] address a multi-item DLS problem, where multiple items share limited production resources. They consider both single-level and multi-level cases and propose a simple three-stage approach that is applicable to both classes of problems. Kim and Lee [46] study a dynamic inbound ordering and shipment scheduling problem for multiple products that are transported from a supplier to a warehouse by common freight containers. They show their problem is NP-hard and present a heuristic algorithm that exploits the properties of an optimal solution. Akbalik *et al*. [47] study a multi-item DLS problem with inventory bounds, and prove that the problem is strongly NP-hard. However, they show that it can be polynomially solved for any fixed number of items under the non-speculative cost assumption. The other studies on production planning and inventory management that relate to our work, see [48–54].

Before we proceed with the details of the multi-product DLS problem with perishable inventory and joint ordering. We summarize the major contributions of this article:

1. We develop an efficient DP algorithm to solve the problem in polynomial time when the number of products is fixed.
2. Based on the monotonicity of order point, we characterize a sufficient condition for a period *t* to be forecast horizon.
3. Using a detailed test bed of instance, we obtain useful managerial insights on the impact of deterioration rate and lifetime of products on the length of forecast horizon. We conclude that (i) as the inventory deterioration rate increases, the length of forecast horizon decreases, then remains invariant; (ii) as the lifetime of products increases, the length of forecast horizon increases, then remains invariant; (iii) for a given deterioration rate, the length of forecast horizon is higher for higher values of joint setup costs; for a given lifetime of products, the length of forecast horizon is higher for higher values of joint setup costs, then remains invariant.

The rest of the paper is organized as follows. We will present our DLS problem with age-dependent stock deterioration rates and inventory cost function and joint ordering in Section 2. In Section 3, we will prove two structural properties and develop a DP algorithm to solve the problem under general time-varying cost structure. We then propose a special instance with no speculative motive in holding inventory. Section 4 establishes forecast horizon results. In section 5, we present computational experience and managerial insights. Section 6 concludes the paper.

## 2. Model formulation

The following notion is used in our model:

*T*: the problem horizon; i.e., the number of periods;
*N*: total number of products;
$d_{t}^{n}$: demand of product *n* in period *t*, 1 ≤ *n* ≤ *N*, 1 ≤ *t* ≤ *T*;
*K*_*t*_: joint setup cost when one or more products are ordered in period *t*, 1 ≤ *t* ≤ *T*;
$\sigma_{t}^{n}$: individual setup cost when product *n* is ordered in period *t*, 1 ≤ *n* ≤ *N*, 1 ≤ *t* ≤ *T*;
$c_{t}^{n}$: unit order cost of product *n* in period *t*, 1 ≤ *n* ≤ *N*, 1 ≤ *t* ≤ *T*;
$h_{it}^{n}$: unit holding inventory cost of product *n* in period *t*, which is ordered in period *i*, 1 ≤ *n* ≤ *N*, 1 ≤ *i* ≤ *t* ≤ *T*;
$x_{t}^{n}$ the amount of product *n* ordered in period *t*, 1 ≤ *n* ≤ *N*, 1 ≤ *t* ≤ *T*;
$z_{it}^{n}$: the amount of the demand of product *n* in period *t* to be satisfied from order in period *i*, 1 ≤ *n* ≤ *N*, 1 ≤ *i* ≤ *t* ≤ *T*;
$y_{it}^{n}$: the amount of inventory of product *n* at the beginning of period *t*, which was ordered in period *i*, 1 ≤ *n* ≤ *N*, 1 ≤ *i* ≤ *t* ≤ *T*;
$\alpha_{it}^{n}$: the fraction of $y_{it}^{n}$ that is lost during period *t*, 1 ≤ *n* ≤ *N*, 1 ≤ *i* ≤ *t* ≤ *T*;

As Hsu [39] points out, regarding deterioration rates and inventory cost, the longer a unit is carried in stock, the faster it may deteriorate and the higher its inventory carrying cost. Therefore, we have the following assumptions:

Assumption 1: $\alpha_{it}^{n}\geq \alpha_{jt}^{n}$, 1 ≤ *n* ≤ *N*; 1 ≤ *i* ≤ *j* ≤ *t* ≤ *T*.
Assumption 2: $h_{it}^{n}\geq h_{jt}^{n}$, 1 ≤ *n* ≤ *N*; 1 ≤ *i* ≤ *j* ≤ *t* ≤ *T*.

Without loss of generality, we assume zero inventory at the beginning of period 1 and at the end of period *T*. We also assume that lead time to satisfy demands is zero.

Define binary variable *δ*(*x*), $\delta (x)=\{\begin{array}{c} 1;\begin{array}{cc} & x>0 \end{array}\\ 0;\begin{array}{cc} & x=0 \end{array} \end{array}$.

We now present the multi-item DLS problem with perishable inventory and joint ordering.


$$\min\sum_{t=1}^{T}\sum_{n=1}^{N}\left[K_{t}\delta (\sum_{n=1}^{N}x_{t}^{n})+\sigma_{t}^{n}\delta (x_{t}^{n})+c_{t}^{n}x_{t}^{n}+\sum_{i=1}^{t}h_{it}^{n}y_{it}^{n}\right]$$

Subject to:

$$x_{t}^{n}-z_{tt}^{n}=y_{tt}^{n}\;\mathrm{for}\;1\leq n\leq N;\;1\leq t\leq T$$ (1)

$$(1-\alpha_{i,t-1}^{n})y_{i,t-1}^{n}-z_{it}^{n}=y_{it}^{n}\;\mathrm{for}\;1\leq n\leq N;\;1\leq i<t\leq T$$ (2)

$$\sum_{i=1}^{t}z_{it}^{n}=d_{t}^{n}\;\mathrm{for}\;1\leq n\leq N;\;1\leq t\leq T$$ (3)

$$x_{it}^{n},y_{it}^{n},z_{it}^{n}\geq 0\;\mathrm{for}\;1\leq n\leq N;\;1\leq i\leq t\leq T$$ (4)

Constraint (1) shows that after using $z_{tt}^{n}$ units out of a total of $x_{t}^{n}$ units order of product *n* to satisfy the demands of product *n* in period *t*, the remaining $y_{tt}^{n}$ units are carried as inventory. Constraint (2) says that after subtracting $z_{it}^{n}$ units from $(1-\alpha_{i,t-1}^{n})y_{i,t-1}^{n}$ to satisfy the demands of product *n* in period *t*, the remaining stock is $y_{it}^{n}$. Constraint (3) means the demands of product *n* in period *t* must be satisfied by ordering product *n* in periods 1 through *t*. Constraint (4) is the non-negative constraint. We will denote this problem as (*MP*).

Define $A_{ii}^{ni}\equiv 1$ and $A_{kt}^{ni}=\frac{1}{\prod_{l=k}^{t-1}(1-\alpha_{il}^{n})}$, for 1 ≤ *n* ≤ *N*; 1 ≤ *i* ≤ *k* < *t* ≤ *T*.

By definition, it is easy to see that

$$A_{kt}^{ni}=A_{kq}^{ni}A_{qt}^{ni},\;\mathrm{for}\;1\leq n\leq N;\;k<q<t.$$

By assumption 1, we have

$$A_{kt}^{ni}\geq A_{kt}^{nj},\;\mathrm{for}\;1\leq n\leq N;\;1\leq i<j\leq k<t\leq T.$$

In addition, to satisfy one unit demand of product *n* (1 ≤ *n* ≤ *N*) in period *t* by an order in period *i* (*i* < *t*), we need to order $A_{it}^{ni}$ units products *n* in period *i* and carry $A_{kt}^{ni}$ units of inventory in each period *k*, where *i* ≤ *k* ≤ *t* − 1.

If $0\leq \alpha_{i,t-1}^{n}<1$ and $\alpha_{it}^{n}=1$, *n* = 1,2, *t* ≥ *i* + 1, we define the lifetime of product *n* is *t* − *i* + 1 periods, denote *m*^*n*^ as the lifetime of product *n*, so *m*^*n*^ = *t* − *i* + 1, if $\alpha_{ii}^{n}=1$, we say the lifetime of product *n* is 1 period.

Note that a period *t* is called an order point (period) of product *n* if $x_{t}^{n}>0$.

## 3. Properties of the optimal solution and DP algorithm

The following theorem implies that there does not exist a polynomial time algorithm for the problem.

**Theorem 1.** Problem (*MP*) is NP-hard in the strong sense.

**Proof.** The proof is similar to other multi-product DLS problems, so its proof is omitted.

In the next subsection, we present some properties for the optimal solution under two assumptions on the cost function, one is general time-varying cost structure, the other is the cost structure with no speculative motive in holding inventory. These properties will be employed later in developing a DP algorithm to solve problem (*MP*), which runs in a polynomial time with a fixed number of products *N*.

### 3.1. General time-varying cost structure

The existing DP algorithm to solve conventional DLS problems typically depends on ZIP, while with age-dependent stock deterioration and inventory cost function, ZIP does not hold under general time-varying cost structure in an optimal solution. This has been demonstrated by single product in Hsu [39]. Now we are ready to prove two structural properties to present a new DP recursion.

**Theorem 2.** There exists an optimal solution Ω* to problem (*MP*) such that: if *i* ≠ *j*, then $(d_{t}^{n}-z_{it}^{n^{*}})(d_{t}^{n}-z_{jt}^{n^{*}})=0$, 1 ≤ *n* ≤ *N*; 1 ≤ *i* ≤ *t*; 1 ≤ *j* ≤ *t*.

**Proof**. Suppose *i* < *j* are two order points of product *n* in the optimal solution Ω*. $c_{i}^{n}A_{it}^{ni}+\sum_{l=i}^{t-1}h_{il}^{n}A_{lt}^{ni}$ represents the total variable order and inventory costs to satisfy one unit demand of product *n* in period *t* by order in period *i*; $c_{j}^{n}A_{jt}^{nj}+\sum_{l=j}^{t-1}h_{jl}^{n}A_{lt}^{nj}$ represents the total variable order and inventory costs to satisfy one unit demand of product *n* in period *t* by order in period *j*. If $c_{i}^{n}A_{it}^{ni}+\sum_{l=i}^{t-1}h_{il}^{n}A_{lt}^{ni}>c_{j}^{n}A_{jt}^{nj}+\sum_{l=j}^{t-1}h_{jl}^{n}A_{lt}^{nj}$, then $z_{jt}^{n^{*}}=d_{t}^{n}$, if not, for any $0<\varepsilon \leq d_{t}^{n}$, assume $z_{jt}^{n^{*}}=d_{t}^{n}-\varepsilon$, $z_{it}^{n^{*}}=\varepsilon$, we have

$$(c_{i}^{n}A_{it}^{ni}+\sum_{l=i}^{t-1}h_{il}^{n}A_{lt}^{ni})\cdot \varepsilon +(c_{j}^{n}A_{jt}^{nj}+\sum_{l=j}^{t-1}h_{jl}^{n}A_{lt}^{nj})\cdot (d_{t}^{n}-\varepsilon )>(c_{j}^{n}A_{jt}^{nj}+\sum_{l=j}^{t-1}h_{jl}^{n}A_{lt}^{nj})\cdot d_{t}^{n},$$

so $z_{jt}^{n^{*}}=d_{t}^{n}$ is true. Similarly, If $c_{i}^{n}A_{it}^{ni}+\sum_{l=i}^{t-1}h_{il}^{n}A_{lt}^{ni}\leq c_{j}^{n}A_{jt}^{nj}+\sum_{l=j}^{t-1}h_{jl}^{n}A_{lt}^{nj}$, then $z_{it}^{n^{*}}=d_{t}^{n}$. Hence we conclude if *i* ≠ *j*, then $(d_{t}^{n}-z_{it}^{n^{*}})(d_{t}^{n}-z_{jt}^{n^{*}})=0$, 1 ≤ *n* ≤ *N*; 1 ≤ *i* ≤ *t*; 1 ≤ *j* ≤ *t*.

Theorem 2 shows that the demand of product *n* in period *t* is satisfied entirely by ordering product *n* in exactly one of periods *i*, 1 ≤ *i* ≤ *t*.

Before presenting Theorem 3, firstly we identify a property of an optimal solution.

**Lemma 1.** Suppose *i* < *j* are two order points of product *n* (1 ≤ *n* ≤ *N*) in the optimal solution Ω^+^ to problem (*MP*) and if $z_{jk}^{n+}=d_{k}^{n}$, for some *j* ≤ *k*, then for any *ε*, $0\leq \varepsilon \leq d_{k}^{n}$, there exists $c_{i}^{n}A_{ik}^{ni}+\sum_{l=i}^{k-1}h_{il}^{n}A_{lk}^{ni}-c_{j}^{n}A_{jk}^{nj}-\sum_{l=j}^{k-1}h_{jl}^{n}A_{lk}^{nj}\geq 0$.

**Proof.** For any constant *ε*, $0\leq \varepsilon \leq d_{k}^{n}$, we modify optimal solution Ω^+^ to solution Ω*, $z_{ik}^{n^{*}}=\varepsilon$, $z_{jk}^{n^{*}}=d_{k}^{n}-\varepsilon$, by definition of $A_{kt}^{ni}$, it is easy to see that in the new solution Ω*, we have $x_{i}^{n^{*}}=x_{i}^{n+}+A_{ik}^{ni}\varepsilon$; $x_{j}^{n^{*}}=x_{j}^{n+}-A_{jk}^{nj}\varepsilon$; $y_{il}^{n^{*}}=y_{il}^{n+}+A_{lk}^{ni}\varepsilon$, *i* ≤ *l* ≤ *k* − 1; $y_{jl}^{n^{*}}=y_{jl}^{n+}-A_{lk}^{nj}\varepsilon$, *j* ≤ *l* ≤ *k* − 1. Denote *V*(Ω^+^) as the total costs of optimal solution Ω^+^ and *V*(Ω*) as the total cost of modified solution Ω*. The perturbed solution can not decrease cost below the optimal solution, so we have *V*(Ω*)−*V*(Ω^+^) ≥ 0, further simplify the above expression, we have:

$(c_{i}^{n}A_{ik}^{ni}+\sum_{l=i}^{k-1}h_{il}^{n}A_{lk}^{ni}-c_{j}^{n}A_{jk}^{nj}-\sum_{l=j}^{k-1}h_{jl}^{n}A_{lk}^{nj})\varepsilon \geq 0$, since $0\leq \varepsilon \leq d_{k}^{n}$, we have:

$$c_{i}^{n}A_{ik}^{ni}+\sum_{l=i}^{k-1}h_{il}^{n}A_{lk}^{ni}-c_{j}^{n}A_{jk}^{nj}-\sum_{l=j}^{k-1}h_{jl}^{n}A_{lk}^{nj}\geq 0$$.

**Theorem 3.** There exists an optimal solution Ω* to problem (*MP*), where if *i* < *j* are two order points of product *n* and $z_{jk}^{n^{*}}=d_{k}^{n}$ for some *k* ≥ *j*, then $z_{it}^{n^{*}}=0$ for each *t*, *k* ≤ *t* ≤ *T*.

**Proof.** Suppose there is an optimal solution Ω^+^ of (*MP*) where *i* < *j* are two order points of product *n* and $z_{jk}^{n+}=d_{k}^{n}$, $z_{it}^{n+}=d_{t}^{n}$ for some *t* > *k* ≥ *j*. We modify the optimal solution Ω^+^ to obtain another feasible solution Ω*, $z_{it}^{n^{*}}=0$, $z_{jt}^{n^{*}}=d_{t}^{n}$. In the modified solution Ω*, we have $x_{i}^{n^{*}}=x_{i}^{n+}-A_{it}^{ni}d_{t}^{n}$; $x_{j}^{n^{*}}=x_{j}^{n+}+A_{jt}^{nj}d_{t}^{n}$; $y_{il}^{n^{*}}=y_{il}^{n+}-A_{lt}^{ni}d_{t}^{n}$, *i* ≤ *l* ≤ *t* − 1; $y_{jl}^{n^{*}}=y_{jl}^{n+}+A_{lt}^{nj}d_{t}^{n}$, *j* ≤ *l* ≤ *t* − 1.

*V*(Ω^+^) represents the costs in the optimal solution Ω^+^, *V*(Ω*) represents the costs in the modified solution Ω*.


$$\begin{array}{l} V(\Omega^{+})-V(\Omega^{*})=c_{i}^{n}A_{it}^{ni}d_{t}^{n}+\sum_{l=i}^{t-1}h_{il}^{n}A_{lt}^{ni}d_{t}^{n}-c_{j}^{n}A_{jt}^{nj}d_{t}^{n}-\sum_{l=j}^{t-1}h_{jl}^{n}A_{lt}^{nj}d_{t}^{n}\\ \begin{array}{cccc} & & & \begin{array}{ccc} & & \;=(c_{i}^{n}A_{it}^{ni}+\sum_{l=i}^{t-1}h_{il}^{n}A_{lt}^{ni}-c_{j}^{n}A_{jt}^{nj}-\sum_{l=j}^{t-1}h_{jl}^{n}A_{lt}^{nj})\cdot d_{t}^{n} \end{array} \end{array}\\ \begin{array}{cccc} & & & \begin{array}{ccc} & & \;= \end{array} \end{array}(c_{i}^{n}A_{it}^{ni}+\sum_{l=i}^{k-1}h_{il}^{n}A_{lt}^{ni}-c_{j}^{n}A_{jt}^{nj}-\sum_{l=j}^{k-1}h_{jl}^{n}A_{lt}^{nj})\cdot d_{t}^{n}+(\sum_{l=k}^{t-1}h_{il}^{n}A_{lt}^{ni}-\sum_{l=k}^{t-1}h_{jl}^{n}A_{lt}^{nj})\cdot d_{t}^{n} \end{array}$$

From assumption 1 and 2, we know $(\sum_{l=k}^{t-1}h_{il}^{n}A_{lt}^{ni}-\sum_{l=k}^{t-1}h_{jl}^{n}A_{lt}^{nj})\cdot d_{t}^{n}\geq 0$.

From lemma 1, we know $(c_{i}^{n}A_{it}^{ni}+\sum_{l=i}^{k-1}h_{il}^{n}A_{lt}^{ni}-c_{j}^{n}A_{jt}^{nj}-\sum_{l=j}^{k-1}h_{jl}^{n}A_{lt}^{nj})\cdot d_{t}^{n}\geq 0$.

So we have *V*(Ω^+^) ≥ *V*(Ω*).

Theorem 3 shows that the inventory stocks are used to satisfy demands with first-in-first-out rule in an optimal solution.

We need to employ the following notations to present a new DP recursion.

Define the set *U*_*m*_ including one or more products, in which that the products have the common last setup period for *t*-period problem. Assume there are *Z*(*t*) common last setup periods for *N* products. By the definition, we know 1 ≤ *m* ≤ *Z*(*t*) ≤ *N*, if *N* products have only one common last setup period, then *Z*(*t*) = 1, if *N* products have *N* different last setup periods, then *Z*(*t*) = *N*.

*V*(*t*) = the minimum costs in a *t*-period problem.
*V*_*n*_(*t*) = the minimum costs for ordering product *i* in a *t*-period problem.
$V(i_{1}^{U_{1}},..,i_{n}^{U_{m}},..,i_{N}^{U_{Z(t)}};q_{1}^{U_{1}},..,q_{n}^{U_{m}},..,q_{N}^{U_{Z(t)}};t)$ = the costs in a *t*-period problem of *N* products in which the last setup for product *n* occurs in period $i_{n}^{U_{m}}$ for satisfying the demands of periods $q_{n}^{U_{m}}$, $q_{n}^{U_{m}}+1$,..,*t*.
$V^{U_{m}}(i_{n}^{U_{m}},q_{n}^{U_{m}},t)$ = the costs in a *t*-period problem for the products that have the common last setup period $i_{n}^{U_{m}}$, and for satisfying the demands of periods $q_{n}^{U_{m}}$, $q_{n}^{U_{m}}+1$,..,*t*.
$i_{nt}^{U_{m}}$ = the period in which the last setup takes place in an optimal solution to *t*-period problem.

Thus, it follows from the definition that

$$\begin{array}{l} V(t)=V(i_{1t}^{U_{1}},..,i_{nt}^{U_{m}},..,i_{Nt}^{U_{Z(t)}};q_{1t}^{U_{1}},..,q_{nt}^{U_{m}},..,q_{Nt}^{U_{Z(t)}};t)\\ \;=\min V(i_{1}^{U_{1}},..,i_{n}^{U_{m}},..,i_{N}^{U_{Z(t)}};q_{1}^{U_{1}},..,q_{n}^{U_{m}},..,q_{N}^{U_{Z(t)}};t) \end{array}$$

Where

$$V(i_{1}^{U_{1}},..,i_{n}^{U_{m}},..,i_{N}^{U_{Z(t)}};q_{1}^{U_{1}},..,q_{n}^{U_{m}},..,q_{N}^{U_{Z(t)}};t)=\min\sum_{m=1}^{Z(t)}V^{U_{m}}(i_{n}^{U_{m}},q_{n}^{U_{m}},t)\;\mathrm{and}$$

$$\begin{array}{l} V_{}^{U_{m}}(i_{n}^{U_{m}},q_{n}^{U_{m}},t)=\sum_{n\in U_{m}}V_{n}(i_{n}^{U_{m}}-1)+K_{i_{n}^{U_{m}}}+\sum_{n\in U_{m}}\sigma_{i_{n}^{U_{m}}}^{n}+c_{i_{n}^{U_{m}}}^{n}\sum_{n\in U_{m}}\sum_{j=q_{n}^{U_{m}}}^{t}A_{i_{n}^{U_{m}},t}^{n,i_{n}^{U_{m}}}d_{j}^{n}\\ \;+\sum_{n\in U_{m}}\sum_{k=i_{n}^{U_{m}}}^{q_{n}^{U_{m}}-1}\sum_{l=q_{n}^{U_{m}}}^{t}h_{i_{n}^{U_{m}},k}^{n}A_{k,l}^{n,i_{n}^{U_{m}}}d_{l}^{n}+\sum_{n\in U_{m}}\sum_{k=q_{n}^{U_{m}}}^{t-1}\sum_{l=k+1}^{t}h_{i_{i}^{U_{m}},k}^{n}A_{kl}^{n,i_{n}^{U_{m}}}d_{l}^{n} \end{array}$$

The period $q_{n}^{U_{m}}$ is defined a division point in the above dynamic programming recursion.

For the definition of *U*_*m*_, since 1 ≤ *m* ≤ *N*, 1 ≤ *t* ≤ *T*, the number of combinations of *U*_*m*_ is *o*(2^*N*^), the value of $V_{}^{U_{m}}(i_{n}^{U_{m}},q_{n}^{U_{m}},t)$ can be determined in *o*(*T*^3^2^*N*^). We conclude that the overall complexity of our DP algorithm is *o*(*T*^3^2^*N*^). This implies that the DP algorithm runs in *O*(*T*^3^), When the number of products *N* is fixed.

### 3.2. No speculative motive cost structure

We observe that in real-world applications, very often that there is no economic incentive for the purchaser to carry inventory if the unit order cost fluctuate slightly. This is a situation known in the DLS literature as no speculative motive in holding inventory. In the context of our problem, it implies the following assumption on the cost parameters: for any *n*, 1 ≤ *n* ≤ *N*,

$$c_{i}^{n}A_{it}^{ni}+\sum_{l=i}^{t-1}h_{il}^{n}A_{lt}^{ni}>c_{t}^{n}\;\forall i<t$$

There are two special cases in the assumptions on the cost parameters: one is that unit order cost of any product *n* is constant for all periods, the other is decreasing.

It is well known that there exists an optimal solution to plenty of DLS problems that satisfies the Zero Inventory Property. In the context of our problem, this property can be restated as follows:

Zero Inventory Property (ZIP). In an optimal solution, $y_{i,t-1}^{n}\cdot x_{t}^{n}=0$ for any 1 ≤ *i* < *t* ≤ *T*; 1 ≤ *n* ≤ *N*.

We found that ZIP even holds with stock deterioration and age-dependent inventory cost function under the assumption of no speculative motive in holding inventory cost structure. In the case of ZIP holds, the vast majority of literature for multi-product DLS models gives the efficient dynamic programming algorithm. In this context, we pass over the DP algorithm under no speculative motive cost structure. Notice that the problem (*MP*) remains NP-hard even with no speculative motive in holding inventory cost structure.

## 4. Forecast horizon results

Define $a_{jt}^{n}=c_{j}^{n}A_{jt}^{nj}+\sum_{l=j}^{t-1}h_{jl}^{n}A_{lt}^{nj}$, 1 ≤ *i* ≤ *t* ≤ *T*, if *j* = *t*, $a_{jj}^{n}=c_{j}^{n}$. Let *m*(*nt*) denote the period with the smallest $a_{jt}^{n}$, 1 ≤ *j* ≤ *t*. Define *P*(*t*) as the *t*-period problem. Let *i*(*nt*) denote the last ordering period and *q*(*nt*) denote the last division point of product *n* in an optimal solution of *P*(*t*). Define *i*(*t*) = min{*i*(1*t*),*i*(2*t*),..,*i*(*Nt*)}, *q*(*t*) = min{*q*(1*t*),*q*(2*t*),..,*q*(*Nt*)}.

**Lemma 2.** In the optimal solution of *P*(*t*), if *i*(*nt*) = *m*(*nt*), 1 ≤ *n* ≤ *N*, then there exists *i*(*n*,*t* + 1) ≥ *i*(*nt*).

**Proof.** Consider the optimal solution of *P*(*t* + 1), if *i*(*n*,*t* + 1) = *t* + 1, 1 ≤ *n* ≤ *N*, then we have *i*(*n*,*t* + 1) > *i*(*nt*); if *i*(*n*,*t* + 1) ≤ *t*, assume *i*(*n*,*t* + 1) < *i*(*nt*) = *m*(*nt*), by the definition of *m*(*nt*) and $a_{jt}^{n}$, we have $a_{i(nt)t}^{n}=a_{m(nt)t}^{n}\leq a_{i(n,t+1)t}^{n}$, since $a_{i(nt),t+1}^{n}=(a_{i(nt)t}^{n}+h_{i(nt)t}^{n})A_{t,t+1}^{ni(nt)}=a_{i(nt),t+1}^{n}=(a_{m(nt)t}^{n}+h_{m(nt)t}^{n})A_{t,t+1}^{nm(nt)}$ and $a_{i(n,t+1),t+1}^{n}=(a_{i(n,t+1),t}^{n}+h_{i(n,t+1),t}^{n})A_{t,t+1}^{ni(n,t+1)}$, we have $a_{i(nt),t+1}^{n}=a_{m(nt),t+1}^{n}\leq a_{i(n,t+1),t+1}^{n}$, this means that producing product *n* in period *i*(*nt*) instead of producing in period *i*(*n*,*t* + 1) to satisfy the demands of product *n* in period *t* + 1 can not increase costs, therefore *i*(*n*,*t* + 1) ≥ *i*(*nt*), 1 ≤ *n* ≤ *N*.

**Lemma 3.** In the optimal solution to *P*(*t*), if *i*(*nt*) = *m*(*nt*) for all *n*, 1 ≤ *n* ≤ *N*, then the *P*(t*) for any *t* + 1 ≤ t* ≤ *n* has an optimal solution with at least one ordering point and one division point for all products in the set {*i*(*t*),*i*(*t*) + 1,..,*t*}.

**Proof.** Consider *P*(t*) with *t* + 1 ≤ t* ≤ *T*, from Lemma 2, we have *i*(nt*) ≥ *i*(*nt*) for all *n*, 1 ≤ *n* ≤ *N*, *t* + 1 ≤ t* ≤ *T*, from the above DP algorithm and the definition of *q*(*nt*), we have *q*(*nf*) ≥ *i*(*nf*), *f* = 1,2,..,*T*. From the definition *i*(*t*) and *q*(*t*), we have *i*(t*) ≥ *i*(*t*) and *q*(t*) ≥ *i*(t*). If *q*(t*) ≥ *t* + 1, we examine the optimum solution of *P*(*q*(t*)−1), the optimum solution of *P*(*q*(t*)−1) is a part of optimum solution of *P*(t*), if *q*(*q*(t*)−1) ≥ *t* + 1, we continue the process until we find a period when the segmental solution has one division period in the interval {*i*(*t*),*i*(*t*) + 1,..,*t*}, since *q*(*nf*) ≥ *i*(*nf*), *f* = 1,2,..,*T*, the segmental solution has one ordering period in the interval {*i*(*t*),*i*(*t*) + 1,..,*t*}, the proof is done. We call the set {*i*(*t*),*i*(*t*) + 1,..,*t*} ordering set.

**Theorem 4.** Consider the optimal solution to *P*(*t*) where *i*(*nt*) = *m*(*nt*) for all *n*, 1 ≤ *n* ≤ *N*, if all optimal solutions to *P*(*r*), *r* ∈ {*i*(*t*)−1,*i*(*t*),..,*t*−1}, have the common first *τ* periods’ decisions, then period *τ* is decision horizon, period *t* is the corresponding forecast horizon.

**Proof**. From lemma 3, any *P*(t*) with t* ≥ *t* has an optimal solution with at least one division point for all products belonging to {*i*(*t*),*i*(*t*)+1,..,*t*}. Thus, solution to at least one of the *P*(*r*), *r* ∈ {*i*(*t*)−1,*i*(*t*),..,*t*−1}, provides a part of solution to the *P*(t*). If all these part of solutions have common first *τ* periods’ decisions, the common first *τ* periods’ decisions must be optimal for the *P*(t*). We call the set {*i*(*t*)−1,*i*(*t*),..,*t*−1} division set. The division set is similar to Ludin and Morton’s regeneration set.

## 5. Computational results

Our test bed is similar to the ones suggested by Dawande *et al*. [27,29] and Bardhan *et al*. [33]. For simplicity, the number of products is set at 2, that is *N* = 2. The function $D_{t+1}^{n}=D_{0}^{n}{\left(G\right)}^{t}$, *n* = 1,2, is used to generate the mean demand in each period, where the parameters *G* captures the character of the growth in demand over time and takes three values: 1.000 (flat), 1.005 (increasing), and 0.995 (decreasing). Once the mean is fixed, the actual demand in a period is generated using the function $D_{t+1}^{n}=D_{0}^{n}{\left(G\right)}^{t}+SD_{0}^{n}\xi$, *n* = 1,2, where *ξ* is the standard normal variate. The parameter *S*, a measure of the variability associated with the demand, takes three values: 0.15, 0.50, and 1.15. For product 1, $D_{0}^{1}$ is set at 8; for product 2, $D_{0}^{2}$ is set at 10. At any time, if a demand generated is less than zero, it is set at zero. The joint setup cost *K*_*t*_ is set at 100, *t* = 1,2,..,*T*, the unit order cost $c_{t}^{n}$ is set at 5 and 3, respectively, *n* = 1,2, *t* = 1,2,..,*T*; without loss of generality, the individual setup cost $\sigma_{t}^{n}$ is set at 0, *n* = 1,2, *t* = 1,2,..,*T*. For each combination of the parameters, we generate 9 instances (to enable the easy identification of the median forecast horizon).

*Text 1*. The lifetime of both product is set to 6, the unit holding inventory costs of product 1 and product 2 are set at $h_{ii}^{1}=1.5$, $h_{i,i+1}^{1}=2$, $h_{i,i+2}^{1}=2.5$, $h_{i,i+3}^{1}=3$, $h_{i,i+4}^{1}=3.5$, $h_{i,i+5}^{1}=+\infty$; $h_{ii}^{2}=2$, $h_{i,i+1}^{2}=2.5$, $h_{i,i+2}^{2}=3$, $h_{i,i+3}^{2}=3.5$, $h_{i,i+4}^{2}=4$, $h_{i,i+5}^{2}=+\infty$. The deterioration rate of both products is set at eight grades as Table 1.

**Table 1:** The grades of deterioration rate.

	$\alpha_{ii}^{1}$; $\alpha_{ii}^{2}$	$\alpha_{i,i+1}^{1}$; $\alpha_{i,i+1}^{2}$	$\alpha_{i,i+2}^{1}$; $\alpha_{i,i+2}^{2}$	$\alpha_{i,i+3}^{1}$; $\alpha_{i,i+3}^{2}$	$\alpha_{i,i+4}^{1}$; $\alpha_{i,i+4}^{2}$	$\alpha_{i,i+5}^{1}$; $\alpha_{i,i+5}^{1}$
Grade 1	0.01; 0.02	0.02; 0.03	0.03; 0.04	0.04; 0.05	0.05; 0.06	1; 1
Grade 2	0.02; 0.03	0.03; 0.04	0.04; 0.05	0.05; 0.06	0.06; 0.07	1; 1
Grade 3	0.10; 0.15	0.20; 0.25	0.30; 0.35	0.40; 0.45	0.50; 0.55	1; 1
Grade 4	0.50; 0.55	0.55; 0.60	0.60; 0.65	0.65; 0.70	0.70; 0.75	1; 1
Grade 5	0.60; 0.65	0.65; 0.70	0.70; 0.75	0.75; 0.80	0.80; 0.85	1; 1
Grade 6	0.70; 0.74	0.75; 0.79	0.80; 0.84	0.85; 0.89	0.90; 0.94	1; 1
Grade 7	0.72; 0.75	0.77; 0.80	0.82; 0.85	0.87; 0.90	0.92; 0.95	1; 1
Grade 8	0.74; 0.76	0.79; 0.81	0.84; 0.86	0.89; 0.91	0.94; 0.96	1; 1

**Fig 1:**
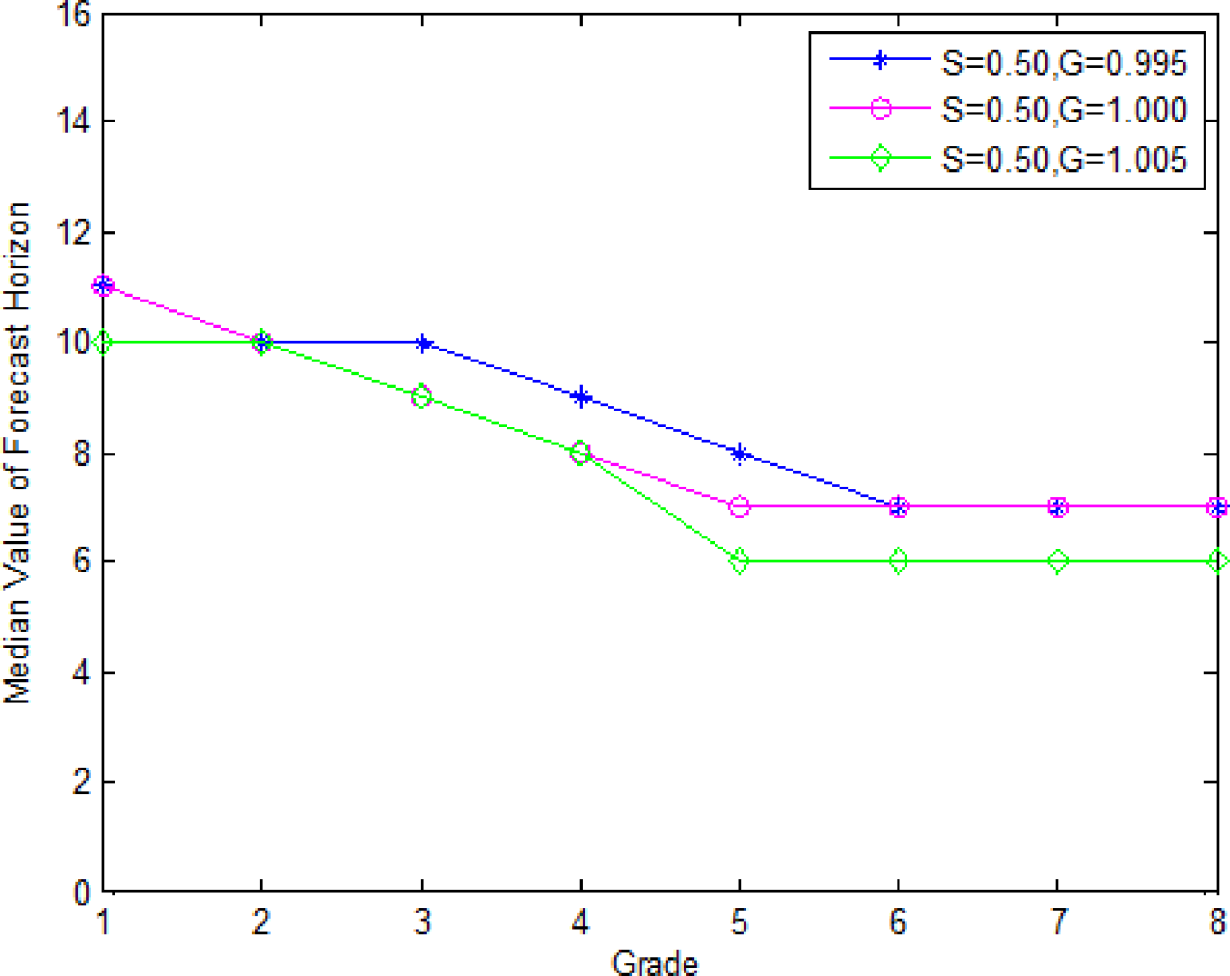
Median forecast horizon as a function of grade and demand growth.

Fig 1 is a plot of the median forecast horizon as a function of deterioration rate for the three values of *G* (0.995, 1.000, 1.005) and *S* set equal to 0.50. For each of three values of *G*, the median forecast horizon decreases with the deterioration rate, then remains constant. When deterioration rate is high, the number of periods covered by the order amount is low, consequently shorter forecast horizon, if the deterioration rate is very high, then lot sizes don’t change, so the forecast horizon remains constant. For a given deterioration rate, the median forecast horizon is larger for smaller values of *G*. When *G* is small, the demands in the future periods get smaller increasingly, as a consequence, there is a worse chance of a lower forecast horizon.

*Text 2*. The lifetime of both products takes seven values: 3, 4, 5, 6, 7, 8, and 9. In order to simplify the calculation program and without loss of generality, the deterioration rate is set to zero within the products’ lifetime. We set the holding costs as follows: for product 1, if *k* + 1 < *m*^1^, *k* is integer, then $h_{i,i+k}^{1}=1.5$, if *k* + 1 ≥ *m*^1^, then $h_{i,i+k}^{1}=+\infty$; for product 2, if *k* + 1 < *m*^2^, then $h_{i,i+k}^{2}=2$, if *k* + 1 ≥ *m*^2^, then $h_{i,i+k}^{2}=+\infty$.

**Fig 2:**
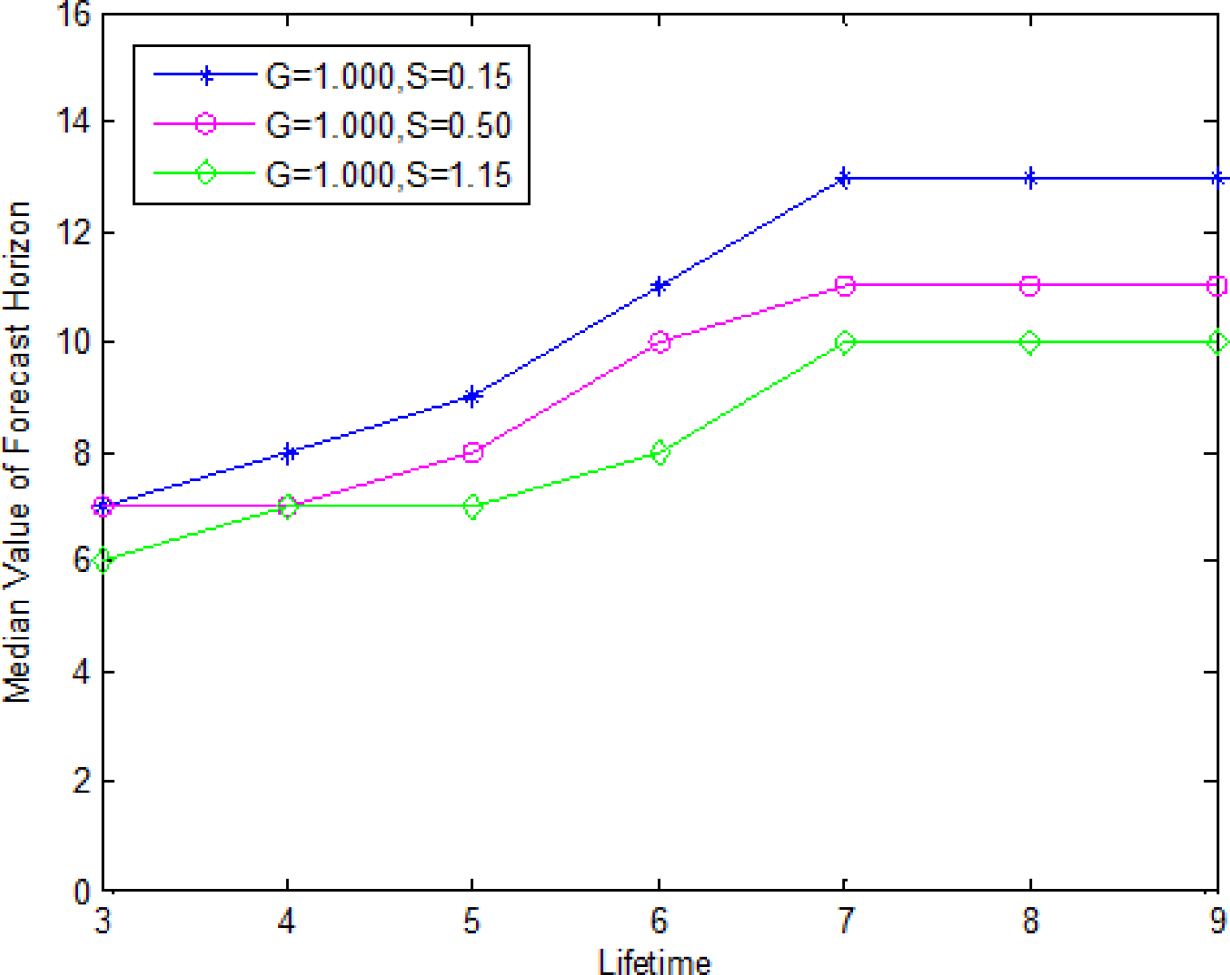
Median forecast horizon as a function of lifetime and demand variability.

Fig 2 is a plot of the median forecast horizon as a function of lifetime of product for the three values of *S* (0.15, 0.50, 1.15) and *G* set equal to 1.000. The median forecast horizon increases with the lifetime, then remains constant. In general, a bigger lifetime of products results in bigger lot sizes, and consequently, a bigger forecast horizon. When the lifetime of products is very large and other parameters are fixed, the perishable products will be the equal of durable products, the forecast horizon can not be affected by the lifetime. For a given lifetime of product, the median forecast horizon is larger for smaller values of *S*. There is an intuitive explanation by Dawande *et al*. [29]: a lower value of *S* indicates a smaller fluctuation in the demand, therefore decreasing the possibility of a large demand, consequently increasing the forecast horizon.

*Text* 3. The joint setup cost takes eight values: 100, 110, 120, 150, 200, 250, 300, and 350. (a) The deterioration rate of both products is set at two grades as follows, the lower grade is $\alpha_{it}^{1}=0.02$, $\alpha_{it}^{2}=0.03$, *i* ≤ *t* ≤ *T*; the higher grade is $\alpha_{it}^{1}=0.70$, $\alpha_{it}^{2}=0.75$, *i* ≤ *t* ≤ *T*. The unit holding costs of both products are set at $h_{it}^{1}=1.5$, $h_{it}^{2}=2$, *i* ≤ *t* ≤ *T*. (b) The lifetime of both products is set at 4, 6, and 9. The deterioration rate is set to zero within the products’ lifetime. We set, for product 1, if *k* + 1 < *m*^1^, $h_{i,i+k}^{1}=1.5$, if *k* + 1 ≥ *m*^1^, $h_{i,i+k}^{1}=+\infty$; for product 2, if *k* + 1 < *m*^2^, $h_{i,i+k}^{2}=2$, if *k* + 1 ≥ *m*^2^, $h_{i,i+k}^{2}=+\infty$.

**Fig 3:**
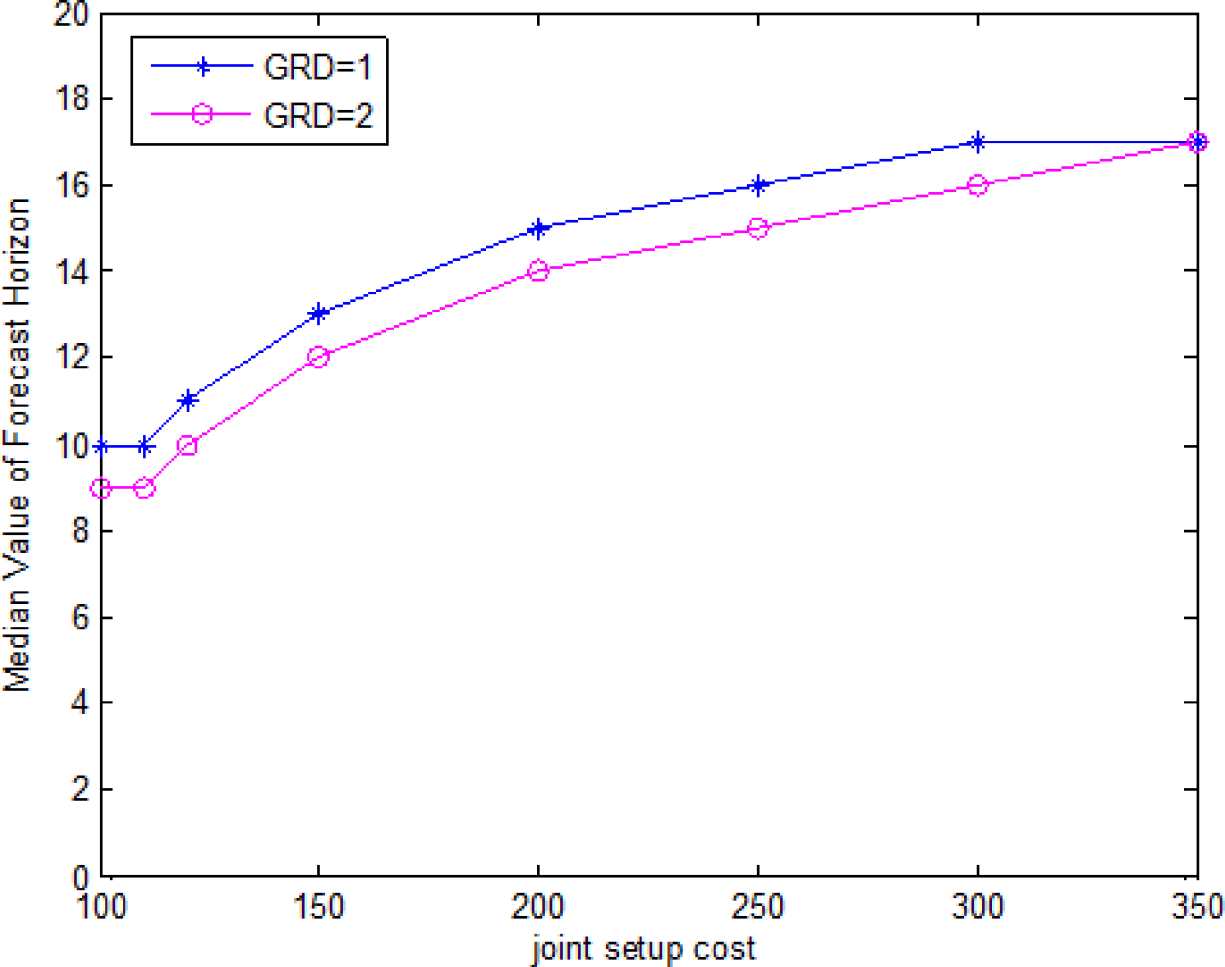
Median forecast horizon as a function of grade and joint setup cost.

**Fig 4:**
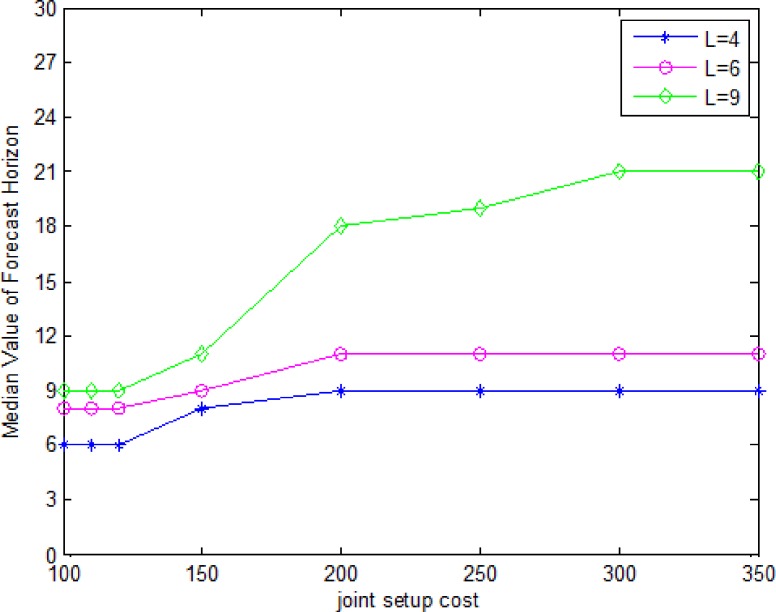
Median forecast horizon as a function of lifetime and joint setup cost.

Fig 3 is a plot of the median forecast horizon as a function of joint setup cost under two grades of deterioration rate (GDR). Fig 4 is a plot of the median forecast horizon as a function of joint setup cost for the three values of lifetime(*L*). For a given joint setup costs, the forecast horizon is lower for a high deterioration rate, is lower for a low lifetime. For a given deterioration rate, the forecast horizon increases with the joint setup cost. Because the higher joint setup costs lead to bigger lot sizes, consequently, longer forecast horizon. For a given lifetime, the forecast horizon increases with the joint setup cost, then remains invariant, since the joint setup costs is enough large, then the lot sizes doesn’t change, hence forecast horizon remains invariant.

## 6. Conclusion and future research

This paper presents a multi-product DLS problem where (i) individual setup cost for each product ordered, joint setup cost is incurred when one or more products are ordered, (ii) stock deterioration rates depend on the age of the stocks and their periods of ordering, (iii) inventory costs depend on the stocks’ ages and the period in which they are carried. To facilitate our analysis, we explore structural properties in an optimal solution and develop a DP algorithm to solve the problem in polynomial time when the number of products is fixed. Based on the monotonicity of ordering point, we establish forecast horizon results that can help the operation manager to decide the precise forecast horizon in a rolling decision-making process. Based on a detailed computational study, we obtain useful managerial insights on the impact of deterioration rate and lifetime of products on the length of forecast horizon. We show that (i) the forecast horizon decreases with an increase in the deterioration rate, then remains invariant; (ii) the forecast horizon increases with an increase in the deterioration rate, then remains invariant; (iii) for a given deterioration rate, the length of forecast horizon is higher for higher values of joint setup costs; for a given lifetime of products, the length of forecast horizon is higher for higher values of joint setup costs, then remains invariant.

One extension of our work would be to study the problem with backloggings or stockouts allowed (e.g., backlogging for single product considered in Hsu [40]). Product substitution, due to the inherent flexibility, has recently been recognized as a resultful instrument to improve the efficiency of multi-product inventory system. The inclusion of one-way product substitution on the extent of joint ordering of multi-product can also provide opportunities for further analysis. Another rich direction is to incorporate capacity constraints including production capacity and warehouse capacity, even though analytical complexity would significantly increase. In such a case, heuristic algorithm may be used to solve the general problem.
